# Spontaneous postnatal complete resolution of an antenatally diagnosed fetal hepatic cyst: A case report

**DOI:** 10.1097/MD.0000000000011133

**Published:** 2018-06-15

**Authors:** Denis Mihai Serban, Costela Serban, Dan Navolan, Ioan Sas

**Affiliations:** a Department of Obstetrics and Gynecology; b Department of Microbiology; c Department of Functional Sciences, “Victor Babes” University of Medicine and Pharmacy, Timisoara, Romania.

**Keywords:** fetal hepatic cyst, spontaneous complete resolution, ultrasound

## Abstract

**Rationale:**

Antenatal diagnosis of a fetal hepatic cyst is rare. Since in most cases the lesion is treated after birth by surgery only a few reports present a complete spontaneous resolution of an antenatally diagnosed hepatic cyst.

**Patient concerns:**

A single hepatic cyst (1.34/1.47 cm) was diagnosed in a fetus at 36 weeks of pregnancy while mother was in labor. After an uneventful vaginal birth, a multidisciplinary evaluation of the newborn confirmed the presence of the cyst.

**Diagnoses:**

Single hepatic cyst

**Interventions:**

Passive approach; periodic ultrasound monitoring of the cyst

**Outcomes:**

The latest ultrasound examination performed at 18 months of age confirmed complete resolution of the cyst. No other medical complications occurred before 18 months of age.

**Lessons:**

The management of such a case depends on the cyst dimensions, the types of involved structure and could require urgent prenatal or neonatal treatment. In our case a complete spontaneous resolution of the hepatic cyst was achieved before 18 months of age.

## 1 Introduction

Although advances in technology have increased the accuracy of antenatal diagnosis of congenital liver anomalies, in utero diagnosis of a fetal hepatic cyst is uncommon. Most of the papers present case reports and series of case reports in which birth is followed by operative treatment.^[1–8]^ Recinos et al present a spontaneous complete resolution of a prenatal diagnosed hepatic cyst.^[9]^ Depending on the dimensions of the cyst and the types of structure that are involved, the management of a hepatic cyst could require an urgent prenatal or neonatal treatment.^[8]^ Short-term complications could be enlargement, obstruction of intrahepatic biliary ducts, pericystic tissue necrosis, infection, and rupture.^[8]^ The malignant transformation is mentioned among long-term complications.^[1]^ The aim of our article was to present the case of a fetus diagnosed with a single hepatic cyst (1.34/1.47 cm) at 36 weeks of pregnancy that presented complete spontaneous resolution until 18 months of age.

## 2 Case report

**Figure 1:**
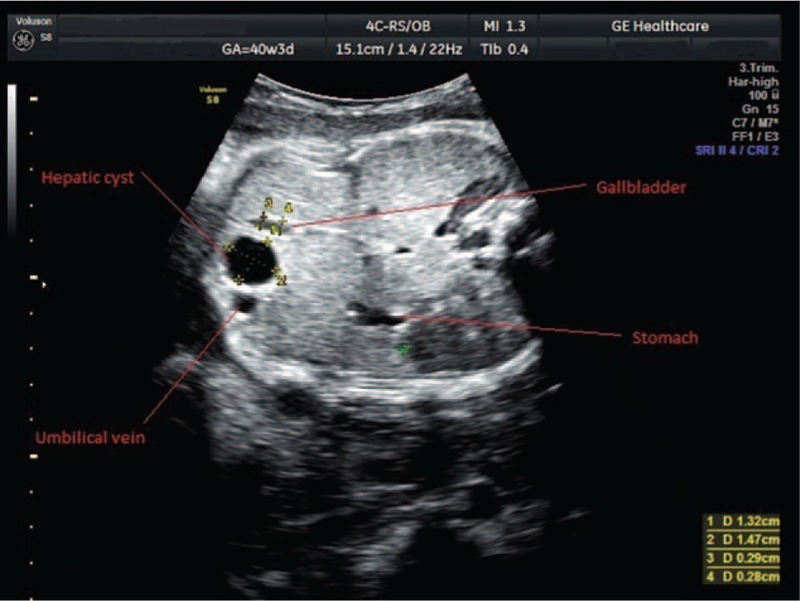
Transabdominal ultrasound of a 36-week-old fetus, showing hepatic cyst.

A 17-year-old woman, gravida 1, para 0, came to our hospital in labor at 36 weeks of gestation for care at birth. Case history revealed that she had not attended any check-ups during her pregnancy. The physical examination showed a 60% to 70% effaced and 3-cm dilated cervix, the fetus had cephalic presentation, and amniotic membranes were intact. Fetal ultrasound showed an isolated anechoic hepatic cyst measuring 1.32/1.47 cm. The cyst was situated in the right anterior abdominal compartment between the gallbladder and the umbilical vein, and at that time it was not certain if the cyst was located in parenchyma or choledocal. Because of the position of the cyst, close to fetal abdominal wall, we suspected that it was a parenchymal cyst (Fig. 1).

**Figure 2:**
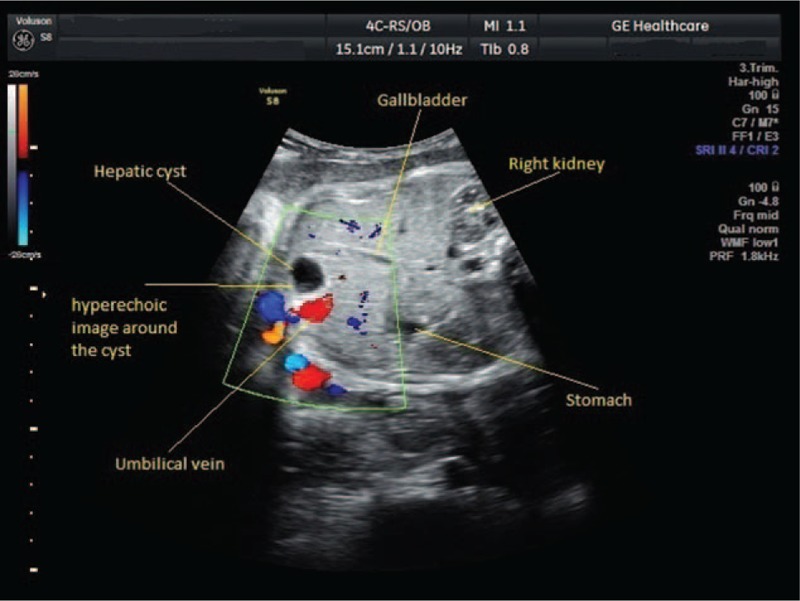
Transabdominal color Doppler ultrasound of a 36-week-old fetus, showing hepatic cyst.

Color Doppler flow imaging did not show any flow in the cystic mass and showed that it was situated to the right of the umbilical vein (Fig. 2).

A female fetus was vaginally delivered in cephalic presentation shortly after her mother was admitted. The weight at birth was 2440 g, and the newborn was diagnosed with low weight for gestational age. The Apgar score value was 8 at 1 minute and 10 at 5 minutes. Unremarkable acid/base, co-oximetry, oxygen status, and electrolytes values from umbilical arterial blood were documented immediately after birth.

At 36 hours after birth the hepatic function panel showed a slightly elevated aspartate aminotransferase level of 43 U/L, slightly greater than the upper limit of normal (37 U/L). The total bilirubin level was of 5.2 mg/dL, corresponding to the low-risk zone at 36 hours after birth, according to the Bhutani nomogram.

**Figure 3:**
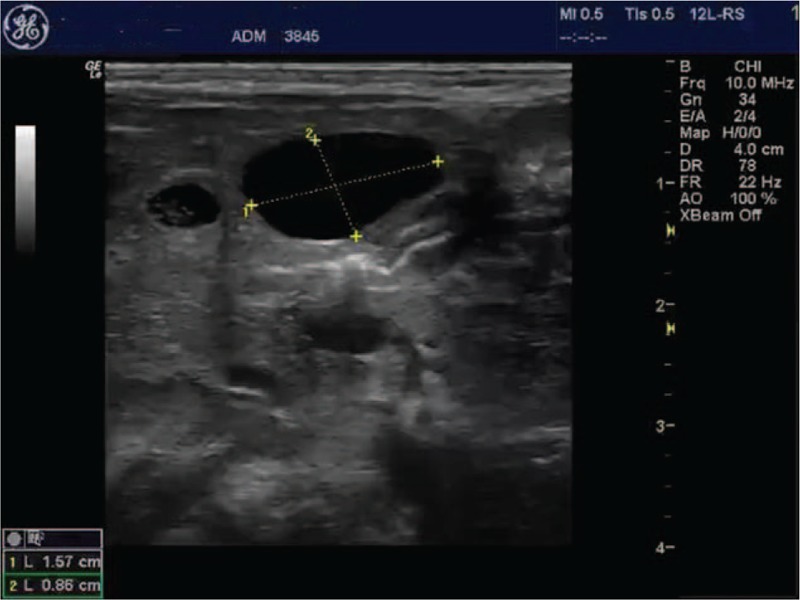
Transabdominal ultrasound immediately after delivery (using a linear probe).

**Figure 4:**
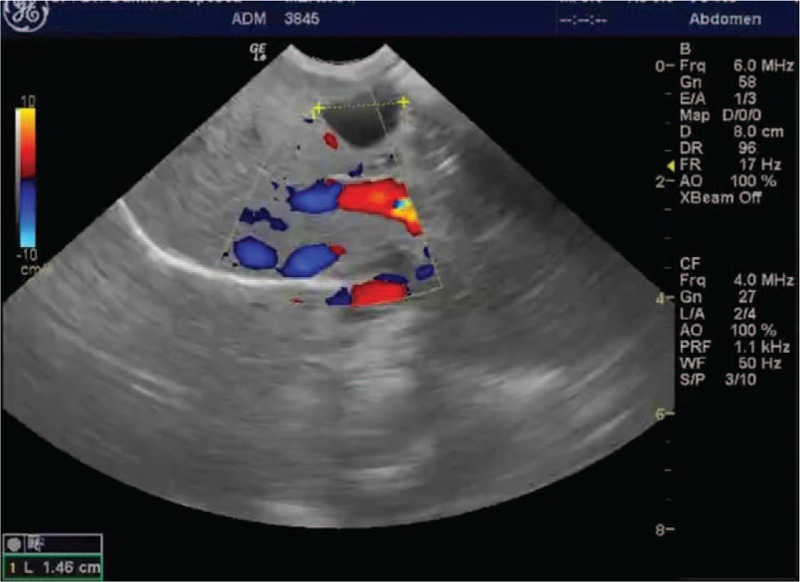
Transabdominal color Doppler ultrasound immediately after delivery (using a convex probe).

The newborn was referred to the pediatric surgery unit, for further multidisciplinary evaluation. The evaluation by abdominal sonography confirmed the presence of a 1.6/0.93 cm intrahepatic cyst situated to the left of the gall bladder, without communication with the gall bladder or the common bile duct. The multidisciplinary team recommended periodic ultrasound monitoring of the cyst (Figs. 3 and 4).

**Figure 5:**
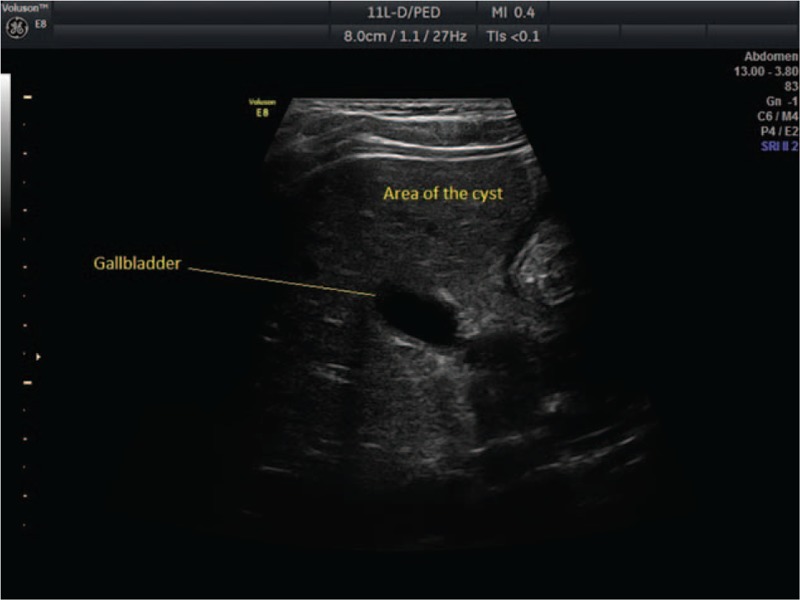
Transabdominal ultrasound showing resolution of liver cyst at 18 months of age.

The baby was reevaluated at 18 months after birth. Laboratory evaluation showed normalization of hepatic function panel and sonography showed complete resolution of the hepatic cyst (Fig. 5).

## 3 Discussion

The overall prevalence of the cystic lesions of the liver is not known, as the majority of cases are asymptomatic.^[1]^ According to Carrim et al the prevalence of hepatic cysts in adults has increased to about 18% due mostly to improved quality of diagnostic imaging.^[10]^ The postnatal prevalence is around 2.5%, but prevalence for antenatal diagnosis has not been reported, since there are only a few cases described in literature.^[2–9]^

According to Paladini differential diagnosis of intrahepatic cysts should be made with: choledocal cyst, gallbladder duplication, or biliary atresia.^[11]^ Differential diagnosis plays a key role in ruling out other pathologies when performing fetal and infant abdominal ultrasound examinations. Depending on the dimensions of the cyst and the types of structure involved, management of the cyst may require urgent prenatal or neonatal treatment.

It has been hypothesized that solitary hepatic cysts are a consequence of failure of intrahepatic ductules to connect with extrahepatic duct during embryogenesis.^[3]^ The incidence is higher in females and the location is favorable to the right hepatic lobe.^[1,2]^ Histological diagnosis of excised congenital hepatic cysts found columnar epithelium in newborns, while atrophic changes of epithelial lining were seen in older children.^[2,3]^

Postnatally, different evolution patterns have been described for antenatally diagnosed hepatic cysts. Several authors proposed, in postnatal period, cystectomy for cases with symptoms^[2,4]^ or cases exhibiting cyst enlargement.^[5,6]^ Cysts were mostly excised via an open abdominal surgery.^[2,4,5]^ For the antenatal approach, in utero percutaneous aspiration was reported.^[6]^ For asymptomatic and non-evolving cysts, a passive approach has been proposed. After a median follow-up of 44 months, Charlesworth et al reported that by using serial postnatal ultrasonography cyst diminution was showed in 4 cases, an enlargement in 1 case, and no dimensional change in 7 cases.^[7]^ Rogers et al reported that out of 5 cysts antenatally diagnosed under sonography surveillance, 4 decreased in size or resolved.^[8]^ More recently, Recinos et al have reported the resorption of an antenatally diagnosed cyst by the seventh month after birth.^[9]^ In our case, complete resolution of the cyst was observed at 18 months after birth.

For asymptomatic hepatic cysts that do not change their size, long-term observation is required to monitor any changes in size or development of pressure symptoms, as noted in various studies.^[3]^ In adults, liver cysts have a good overall prognosis, and complications are rare.^[12]^

In conclusion, antenatally diagnosed fetal hepatic cysts, although rare, do occur. Yet, with the latest advances in imaging techniques, obstetricians are able to diagnose such anomalies during antenatal period, simply by performing a fetal ultrasound. The management of these cases depends on the dimensions of the cyst and the types of structure involved. In our case a complete spontaneous resolution of the hepatic cyst was achieved before the age of 18 months. No other medical complications occurred until this age.

## Acknowledgments

We extend our gratitude to the team in the Gynecology and Obstetrics unit as well as to the neonatologist and pediatric surgeons for their support and interdisciplinary approach.

## Author contributions

**Conceptualization:** Denis Mihai Serban.

**Investigation:** Denis Mihai Serban.

**Writing – original draft:** Denis Mihai Serban, Costela Serban.

**Writing – review & editing:** Dan Navolan, Ioan Sas.
